# Clazosentan Administration Was Not Associated with Intracranial Pressure Elevation in Aneurysmal Subarachnoid Hemorrhage: A Retrospective Study with Direct ICP Monitoring

**DOI:** 10.3390/jcm15176855

**Published:** 2026-09-04

**Authors:** Yo Nishimoto, Tadashi Miura, Ken Shinno, Yongran Yanase, Nao Kagimoto, Shota Nishimoto, Satoru Hayashi, Hitoshi Fukuda

**Affiliations:** 1 Department of Neurosurgery, Chikamori Hospital, Kochi 780-8522, Japan; miuratadashi7222@gmail.com (T.M.); yongran.12k04.ysw@gmail.com (Y.Y.); n.olieve.ek57@gmail.com (N.K.); ns7566u@gmail.com (S.N.); nshayashi@chikamori.com (S.H.); 2 Department of Neurosurgery, Kochi Health Science Center, Kochi 781-8555, Japan; k.shinno.1017@gmail.com; 3 Department of Neurosurgery, Kochi Medical School Hospital, Kochi 783-8505, Japan

**Keywords:** subarachnoid hemorrhage, clazosentan, intracranial pressure, cerebral perfusion pressure, vasospasm, delayed cerebral ischemia, direct ICP monitoring, neurocritical care

## Abstract

**Background/Objectives**: Clazosentan reduces cerebral vasospasm and delayed cerebral ischemia following aneurysmal subarachnoid hemorrhage (aSAH). However, concerns that clazosentan may cause cerebral edema and increase intracranial pressure (ICP) have discouraged its use in patients with poor-grade aSAH. **Methods**: This single-center retrospective study included 59 patients with aSAH who underwent continuous direct ICP monitoring, of whom 23 received clazosentan and 24 received fasudil. Most clazosentan-treated patients had poor-grade aSAH (WFNS 4–5, 95.7%). The primary outcome was the change in mean daily ICP in the clazosentan group between the pre-treatment and full treatment periods, with a secondary analysis restricted to the first 7 days and stratified by the presence of intracerebral hematoma (ICH). Cerebral perfusion pressure (CPP) changes were assessed as secondary outcomes, and the fasudil group served as an exploratory comparator. **Results**: Among 21 clazosentan-treated patients available for primary analysis, median ICP did not change significantly between the pre-treatment and treatment periods (6.4 vs. 8.4 mmHg; median ΔICP +0.5 mmHg, 95% CI −1.74 to +2.64; *p* = 0.708), a finding consistent in the first 7 days and across subgroups by ICH status. In contrast, CPP increased significantly between the pre-treatment and treatment periods (69.1 vs. 84.5 mmHg; median ΔCPP +14.0 mmHg, 95% CI +8.93 to +17.44; *p* < 0.001). **Conclusions**: In this retrospective single-center cohort, no statistically significant ICP elevation was observed during clazosentan administration under standard neurosurgical management. Although the small sample size limits statistical power and a false-negative result cannot be excluded, these findings may help inform clinical decision-making regarding clazosentan use in patients with poor-grade aSAH.

## 1. Introduction

Aneurysmal subarachnoid hemorrhage (aSAH) is a life-threatening cerebrovascular event associated with high morbidity and mortality [1]. Cerebral vasospasm is one of the major causes of delayed cerebral ischemia (DCI) following aSAH, contributing significantly to poor neurological outcomes [2]. Clazosentan, a selective endothelin-1 type A receptor antagonist, has been demonstrated to suppress vasospasm and reduce DCI-related morbidity and mortality [3,4,5,6,7]. Following approval in Japan in April 2022, clazosentan has been increasingly adopted in clinical practice for patients undergoing surgical clipping or endovascular treatment of ruptured intracranial aneurysms.

Clazosentan has been associated with fluid retention, most notably pleural effusion and pulmonary edema [3,4,5,7]. Cerebral edema has also been reported following clazosentan administration [3,7,8]. In aSAH patients, cerebral edema is a well-recognized complication that occurs more frequently in those with poor neurological grades [9], and the resulting elevation in intracranial pressure (ICP) has been established as an independent predictor of poor functional outcome and increased mortality [10,11]. This concern has likely contributed to the cautious use of clazosentan, particularly in patients with poor-grade aSAH or concomitant intracerebral hematoma (ICH)—the very patients who may benefit most from vasospasm prophylaxis. However, no study has directly evaluated the effect of clazosentan on ICP. The aim of the present study was to examine changes in ICP during clazosentan administration using continuous direct ICP monitoring.

## 2. Materials and Methods

### 2.1. Study Design and Setting

This was a single-center, retrospective observational study in patients with aSAH who underwent continuous direct ICP monitoring. Clazosentan became available only after its approval in Japan in April 2022 and was preferentially administered to patients considered suitable for its use. In contrast, fasudil remained in use throughout the study period and, after the approval of clazosentan, was preferentially administered to patients considered less suitable for clazosentan. As a result, the two groups differed systematically in patient characteristics. Direct between-group comparisons of ICP were therefore not performed. Instead, the primary analysis was based on a within-group before-and-after comparison of ICP in the clazosentan group, with each patient serving as their own control. The fasudil group was included as an exploratory comparator to examine whether any observed cerebral perfusion pressure (CPP) changes were specific to clazosentan, and the group receiving no vasospasm prophylaxis was analyzed descriptively. Clazosentan-treated patients were enrolled from March 2023 to December 2025, and fasudil-treated patients from January 2010 to December 2025; patients receiving neither agent were included from the full study period. The study was approved by the Institutional Review Board of Chikamori Hospital (approval number: 1046), and the requirement for individual informed consent was waived owing to the retrospective nature of the study.

### 2.2. Patient Selection

Inclusion criteria were: (1) diagnosis of aSAH confirmed by computed tomography angiography or digital subtraction angiography, and (2) continuous direct ICP monitoring performed during the acute phase of illness. A total of 59 patients met these criteria and were included in the analysis. Of these, 23 received clazosentan, 24 received fasudil, and 12 received no vasospasm prophylaxis. Two patients in the clazosentan group were excluded from the primary analysis because no ICP data were available during the treatment period, leaving 21 patients. In the fasudil group, 8 patients were excluded from the exploratory analysis because ICP data were insufficient during the pre-treatment or treatment period, leaving 16 patients. In the no-vasospasm-prophylaxis group, 1 patient with insufficient ICP recordings was excluded from the ICP-based descriptive analysis, leaving 11 patients.

### 2.3. Perioperative Management

Blood pressure was managed according to a standardized institutional protocol, in which the systolic blood pressure target was maintained below 140 mmHg in the early postoperative period and relaxed to an upper limit of 160–180 mmHg from approximately postoperative day 3–4 onward, corresponding to the vasospasm risk period. Decompressive craniectomy was performed in patients presenting with, or at risk of, impending cerebral herniation based on clinical and radiological assessment, and this indication was applied consistently across all groups. Other interventions that may influence ICP—including sedation, osmotherapy, cerebrospinal fluid drainage, and mechanical ventilation—were not standardized and were determined at the discretion of the treating physicians in accordance with institutional practice.

### 2.4. ICP Monitoring

The decision to initiate ICP monitoring was made at the discretion of the treating physician, without predefined criteria. In practice, ICP monitoring was more likely to be performed in patients with poor-grade aSAH. ICP was measured continuously using the Codman MicroSensor ICP transducer (Integra LifeSciences, Plainsboro, NJ, USA). In patients who underwent craniotomy, the sensor was inserted directly into the brain parenchyma at the time of surgery. In patients treated with endovascular coiling, the sensor was introduced into the brain parenchyma through a burr hole created for external ventricular drainage. Continuous ICP monitoring was initiated upon admission to the intensive care unit. In patients who demonstrated clinical stability with consistently normal ICP values, the sensor was removed prior to day 14 at the discretion of the treating physician. ICP and CPP were recorded at 1–2 h intervals during continuous monitoring. Values recorded outside the regular interval in response to acute clinical changes were also included. Daily mean ICP and CPP values were calculated from all available recordings for each day. The median duration of ICP monitoring was 10 days (IQR 6–15) in the clazosentan group and 9 days (IQR 4–11) in the fasudil group (*p* = 0.231). ICP sensor removal prior to day 14 occurred in 15 clazosentan-treated patients (65.2%) and 21 fasudil-treated patients (87.5%; *p* = 0.093). Cerebral perfusion pressure (CPP) was calculated as mean arterial pressure (MAP) minus ICP (CPP = MAP − ICP) and recorded continuously alongside ICP. Although MAP was continuously measured, it was not separately recorded in the dataset.

### 2.5. Clazosentan Administration

In accordance with the approved Japanese prescribing information, clazosentan was administered at a dose of 300 mg dissolved in 500 mL of normal saline and delivered by continuous intravenous infusion at a rate of 17 mL/hour (10 mg/hour). Treatment was initiated in the early postoperative period and continued until day 14 after aSAH onset. In the present cohort, clazosentan was initiated at a median of day 2 (IQR 2–3) after aSAH onset, with a median treatment duration of 12 days (IQR 12–13).

### 2.6. Fasudil Administration

Fasudil is a Rho-kinase inhibitor approved in Japan for the prevention of cerebral vasospasm following aSAH [12]. In accordance with the approved Japanese prescribing information, fasudil was administered at a dose of 30 mg diluted in 50–100 mL of electrolyte or glucose solution, infused intravenously over approximately 30 min three times daily. Treatment was initiated in the early postoperative period and continued for up to 2 weeks. In the present cohort, fasudil was initiated at a median of day 4 (IQR 3–4) after aSAH onset, with a median treatment duration of 11 days (IQR 10–12).

### 2.7. Data Collection

The following variables were collected from medical records: patient age, sex, WFNS grade, presence of ICH on admission CT, treatment modality (surgical clipping or endovascular treatment), performance of decompressive craniectomy, placement of cerebrospinal fluid (CSF) drainage (external ventricular drainage or spinal drainage), and drug administered (clazosentan, fasudil, or no vasospasm prophylaxis). Drug start and finish days were recorded as days after aSAH onset (onset = day 0). Daily mean ICP and CPP values were collected from day 0 until ICP sensor removal. Functional outcome was assessed by the modified Rankin Scale (mRS) at the most recent clinical follow-up (median follow-up, 70 days; IQR 40–97 days from aSAH onset), and in-hospital mortality was defined as an mRS of 6 recorded during the index hospitalization.

### 2.8. Outcome Measures

The primary outcome was the change in mean daily ICP between the pre-treatment period (day 0 to the day before drug initiation) and the treatment period (from drug initiation to drug completion, or until ICP sensor removal if earlier) in the clazosentan group. A secondary analysis was performed restricting the treatment period to the first 7 days after clazosentan initiation, corresponding to the period of greatest risk for fluid retention-related complications [4]. Additional outcomes included: (1) CPP change in the clazosentan group across both the full treatment period and the first 7 days; (2) subgroup analyses of ICP and CPP changes stratified by ICH status; (3) exploratory analyses of ICP and CPP changes in the fasudil group; and (4) the association between mean ICP or CPP and in-hospital mortality across all ICP-monitored patients.

### 2.9. Statistical Analysis

Continuous variables are presented as median and interquartile range (IQR). The Wilcoxon signed-rank test was used for paired comparisons of ICP and CPP between the pre-treatment and treatment periods within each group. Between-group comparisons of continuous variables were performed using the Mann–Whitney U test, and categorical variables using Fisher’s exact test. A two-tailed *p*-value of less than 0.05 was considered statistically significant. For Wilcoxon signed-rank tests, effect sizes were reported as rank-biserial correlations (r), and location shifts were estimated using the Hodges–Lehmann estimator with corresponding 95% confidence intervals.

Missing daily ICP and CPP values resulting from early sensor removal were not imputed. Analyses were based on the available daily measurements for each patient. Early sensor removal most commonly reflected clinical improvement or patient death. A post hoc power analysis was performed using a normal approximation for the Wilcoxon signed-rank test. No adjustment for multiple comparisons was applied. The primary outcome was pre-specified; all other analyses were exploratory.

All statistical analyses were performed using Python (version 3.12; Python Software Foundation) with the SciPy library (version 1.17.1). During the preparation of this manuscript, the authors used Claude (Anthropic, version claude-sonnet-4-6) to support statistical analysis and figure preparation. All outputs were reviewed and verified by the authors, who take full responsibility for the content of this work.

## 3. Results

### 3.1. Patient Characteristics

**Figure 1:**
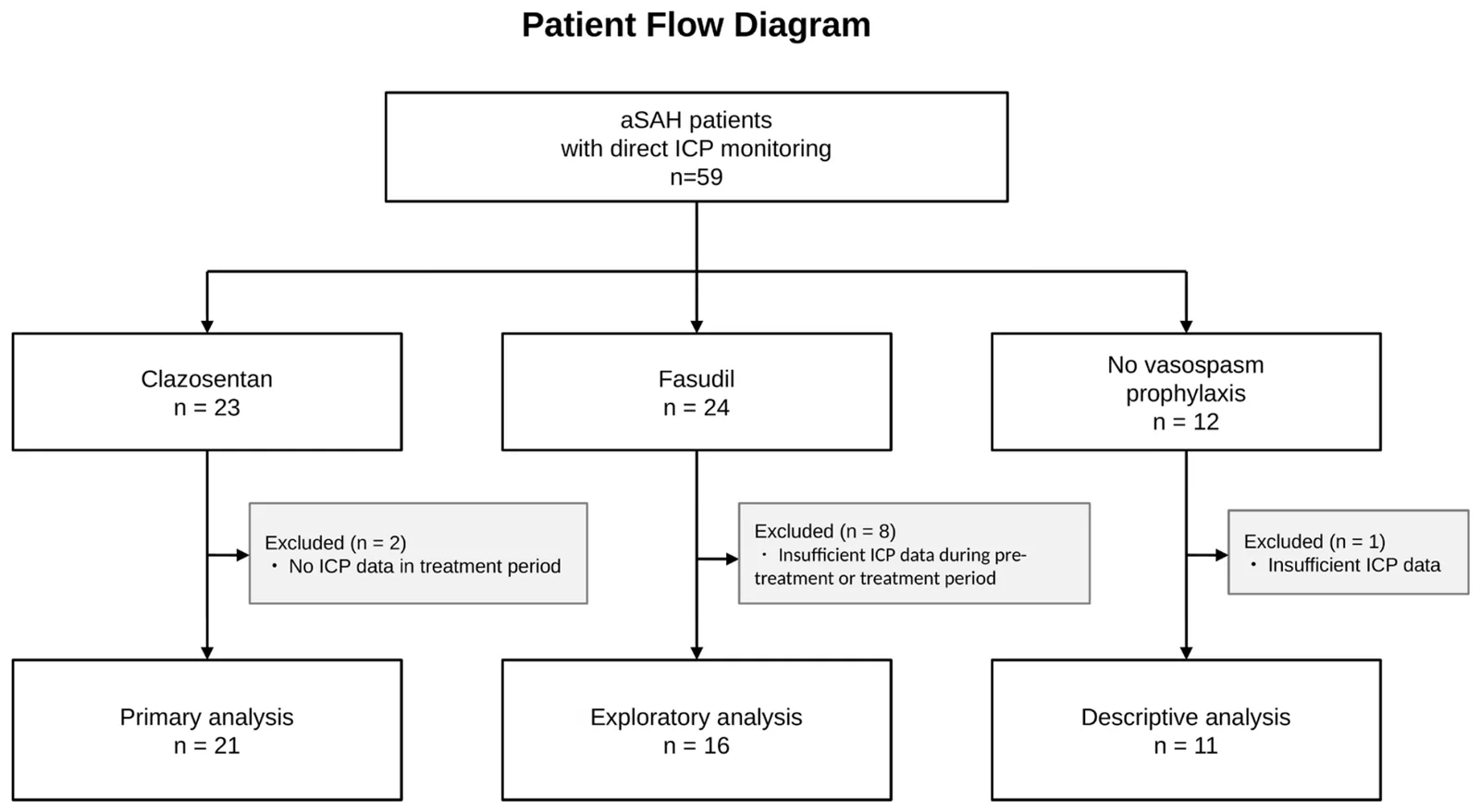
Patient flow diagram. Of 59 patients who underwent continuous direct ICP monitoring, 21 clazosentan-treated patients were available for the primary analysis, 16 fasudil-treated patients for the exploratory analysis, and 11 patients receiving no vasospasm prophylaxis for the descriptive analysis. ICP, intracranial pressure; CPP, cerebral perfusion pressure; aSAH, aneurysmal subarachnoid hemorrhage.

A total of 59 patients with aSAH who underwent continuous direct ICP monitoring were included. Of these, 23 received clazosentan, 24 received fasudil, and 12 received no vasospasm prophylaxis. The patient flow is shown in Figure 1. Patient characteristics are summarized in Table 1.

In the clazosentan group, the median age was 66 years (IQR 58–74), and 16 patients (69.6%) were female. WFNS grade 4 or 5 was present in 22 patients (95.7%). ICH was present in 8 patients (34.8%), and decompressive craniectomy was performed in 7 (30.4%). Surgical clipping was performed in 12 patients (52.2%) and endovascular treatment in 11 (47.8%). Clazosentan was initiated at a median of day 2 (IQR 2–3) after aSAH onset, with a median treatment duration of 12 days (IQR 12–13). There were no in-hospital deaths in this group.

In the fasudil group, the median age was 70 years (IQR 54–76), and 17 patients (70.8%) were female. WFNS grade 4 or 5 was present in 18 patients (75.0%). ICH was present in 16 patients (66.7%), and decompressive craniectomy was performed in 10 (41.7%). Surgical clipping was performed in 21 patients (87.5%) and endovascular treatment in 3 (12.5%). Fasudil was initiated at a median of day 4 (IQR 3–4), with a median treatment duration of 11 days (IQR 10–12). In-hospital mortality was 2 of 24 patients (8.3%).

Comparison between the clazosentan and fasudil groups revealed no significant differences in age (*p* = 0.725), sex (*p* = 1.000), WFNS grade 4–5 (*p* = 0.097), or decompressive craniectomy (*p* = 0.547). However, the fasudil group had a significantly higher rate of ICH (66.7% vs. 34.8%, *p* = 0.042) and a higher proportion of surgical clipping (87.5% vs. 52.2%, *p* = 0.011), reflecting differences in treatment selection between the two cohorts.

In the no vasospasm prophylaxis group (*n* = 12), WFNS grade 5 was present in 10 patients (83.3%), and in-hospital mortality was 11 of 12 patients (91.7%), reflecting the extreme severity of this subgroup.

### 3.2. ICP Changes Before and During Clazosentan Administration

**Figure 2:**
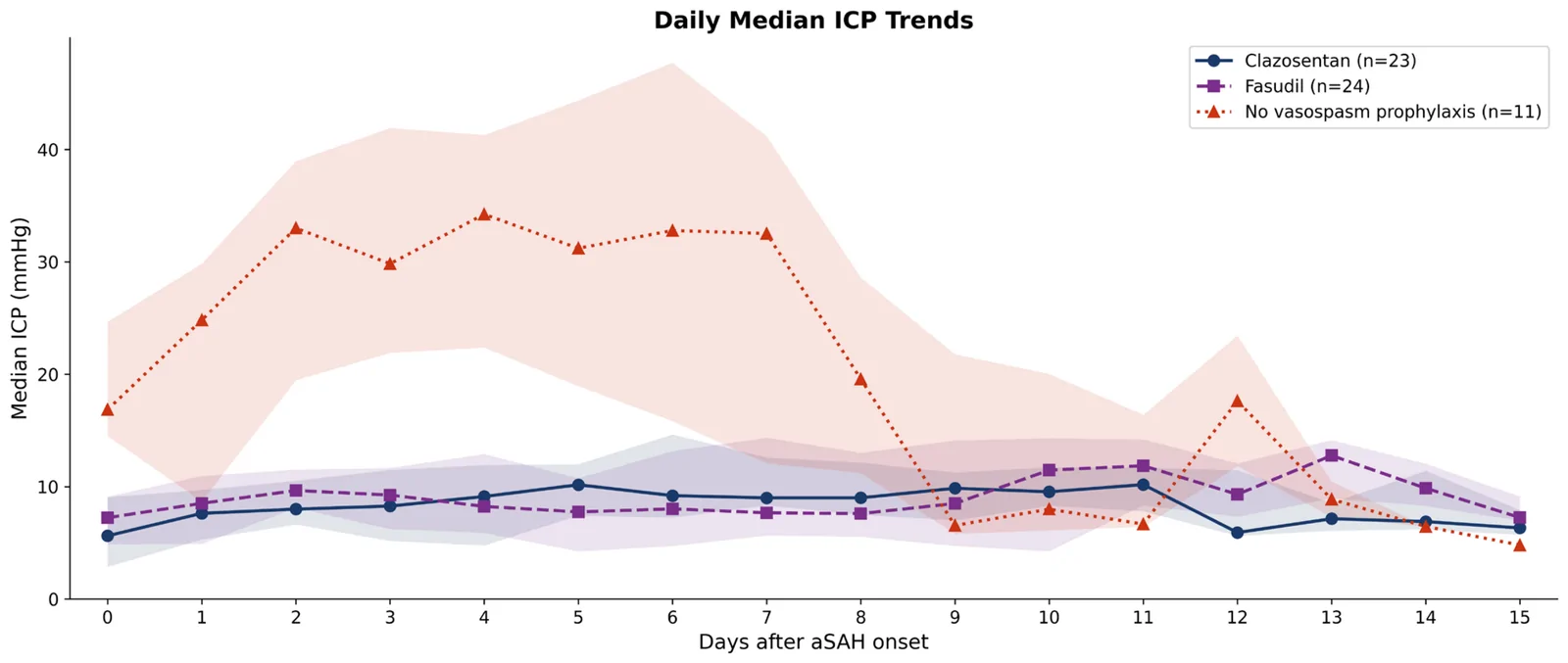
Daily median ICP trends by treatment group. Median daily ICP values from day 0 to day 15 after aSAH onset in the clazosentan group (*n* = 23, solid line), fasudil group (*n* = 24, dashed line), and no vasospasm prophylaxis group (*n* = 11, dotted line), using all available daily ICP recordings regardless of data completeness during the pre-treatment or treatment period. Shaded areas represent interquartile ranges. aSAH, aneurysmal subarachnoid hemorrhage; ICP, intracranial pressure.

The daily median ICP trends across all three treatment groups are shown in Figure 2.

**Figure 3:**
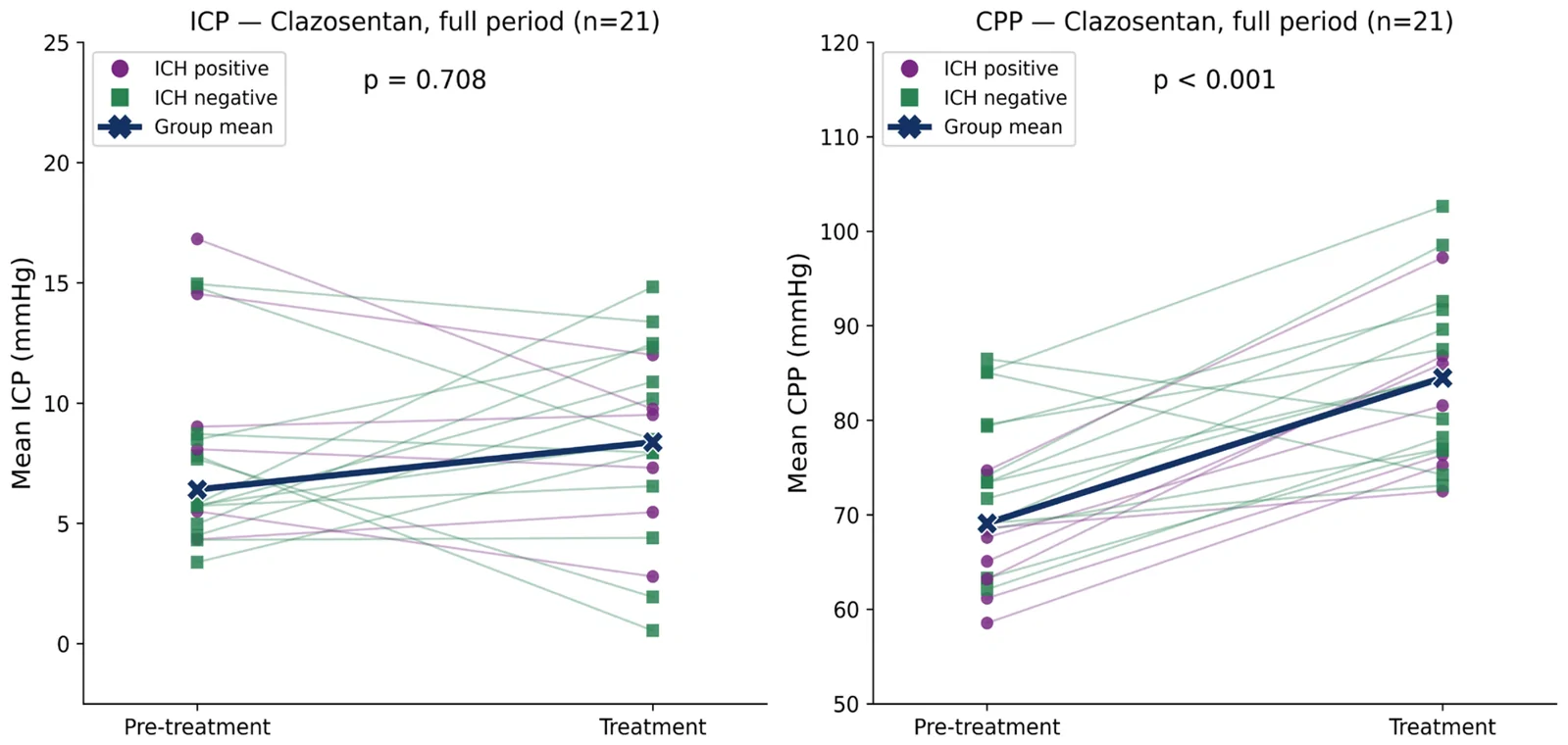
ICP and CPP changes between the pre-treatment and treatment periods (full clazosentan treatment period). Individual changes in mean intracranial pressure (ICP; (**left panel**)) and cerebral perfusion pressure (CPP; (**right panel**)) between the pre-treatment and treatment periods (full clazosentan treatment period) in 21 patients. Each line represents one patient. Purple circles indicate ICH-positive patients and green squares indicate ICH-negative patients. The dark blue X symbol represents the group mean. Statistical comparison was performed using the Wilcoxon signed-rank test. ICH, intracerebral hemorrhage; ICP, intracranial pressure; CPP, cerebral perfusion pressure.

Of the 23 clazosentan-treated patients, 2 were excluded from the primary analysis, resulting in 21 patients available for the primary analysis. The median ICP before clazosentan initiation was 6.4 mmHg (IQR 5.5–8.7 mmHg), and the median ICP during the full treatment period was 8.4 mmHg (IQR 6.6–10.9 mmHg), yielding a median ΔICP of +0.5 mmHg (IQR −2.5 to +3.8 mmHg; *p* = 0.708; rank-biserial correlation r = 0.10; 95% CI −1.74 to +2.64 mmHg). No patient in the clazosentan group had a mean ICP exceeding 20 mmHg during the monitoring period (Figure 3).

Subgroup analyses stratified by ICH status showed no significant ICP elevation: ICH-positive patients (*n* = 7) showed a median ΔICP of −0.8 mmHg (95% CI −4.89 to +1.55; *p* = 0.375), and ICH-negative patients (*n* = 14) showed a median ΔICP of +1.7 mmHg (95% CI −1.72 to +4.55; *p* = 0.426).

**Figure 4:**
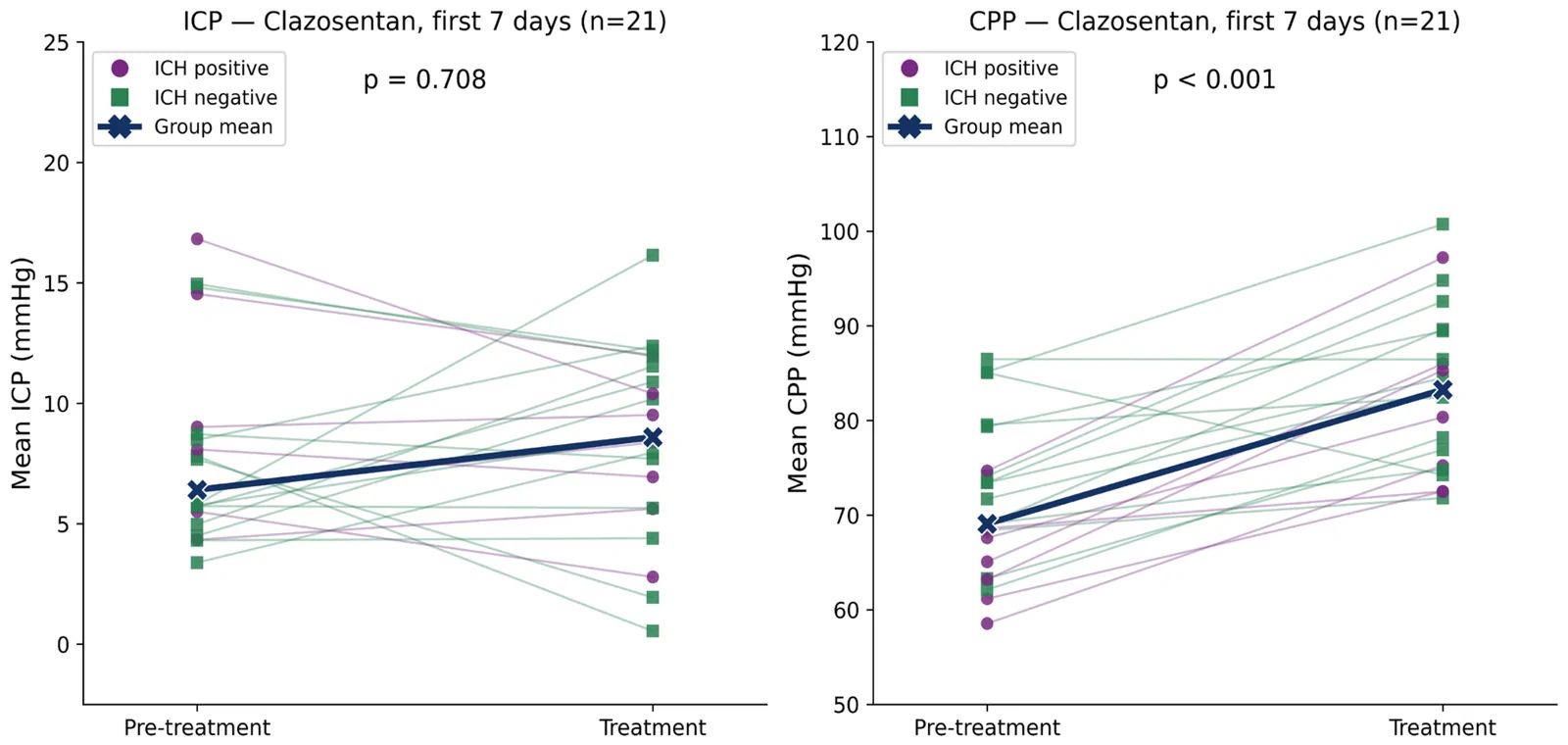
ICP and CPP changes between the pre-treatment period and the first 7 days of clazosentan administration. Individual changes in mean ICP (left panel) and CPP (right panel) between the pre-treatment period and the first 7 days of clazosentan administration in 21 patients. The 7-day window was selected based on CONSCIOUS-1 trial data indicating that pulmonary complications occur predominantly within this period. Symbols and colors are as described in Figure 3. Statistical comparison was performed using the Wilcoxon signed-rank test.

A secondary analysis restricted to the first 7 days after clazosentan initiation—the period most susceptible to fluid retention-related complications based on the CONSCIOUS-1 trial—likewise demonstrated no significant ICP change (median 8.6 mmHg vs. 6.4 mmHg; median ΔICP +0.09 mmHg, IQR −2.61 to +3.90 mmHg; 95% CI −1.78 to +2.59 mmHg; *p* = 0.708) (Figure 4).

### 3.3. Safety Outcomes in the Clazosentan Group

Fluid retention complications—defined as pleural effusion, pulmonary edema, or ascites—were observed in 12 of 23 clazosentan-treated patients (52.2%). Early discontinuation of clazosentan prior to day 14 occurred in 4 patients (17.4%), all of whom had documented fluid retention complications. Radiographic vasospasm was observed in 8 patients (34.8%), and delayed cerebral ischemia confirmed by MRI-documented infarction occurred in 4 patients (17.4%). Diuretic therapy was administered in 13 patients (56.5%).

### 3.4. CPP Changes Before and During Clazosentan Administration

CPP increased significantly during clazosentan administration. The median CPP before treatment was 69.1 mmHg (IQR 65.1–74.7 mmHg), rising to 84.5 mmHg (IQR 76.8–89.6 mmHg) during the full treatment period (median ΔCPP +14.0 mmHg, IQR +8.5 to +19.2 mmHg; rank-biserial correlation r = 0.92; 95% CI +8.93 to +17.44 mmHg; *p* < 0.001). This CPP elevation was significant in both ICH-positive (ΔCPP +16.7 mmHg, 95% CI +8.93 to +23.08; *p* = 0.016) and ICH-negative patients (ΔCPP +12.7 mmHg, 95% CI +4.19 to +16.47; *p* = 0.002). In the secondary analysis restricted to the first 7 days, CPP elevation remained significant (median ΔCPP +12.75 mmHg, IQR +5.72 to +19.16 mmHg; 95% CI +8.08 to +16.32 mmHg; *p* < 0.001).

### 3.5. Exploratory Analysis: ICP and CPP Changes in the Fasudil Group

**Figure 5:**
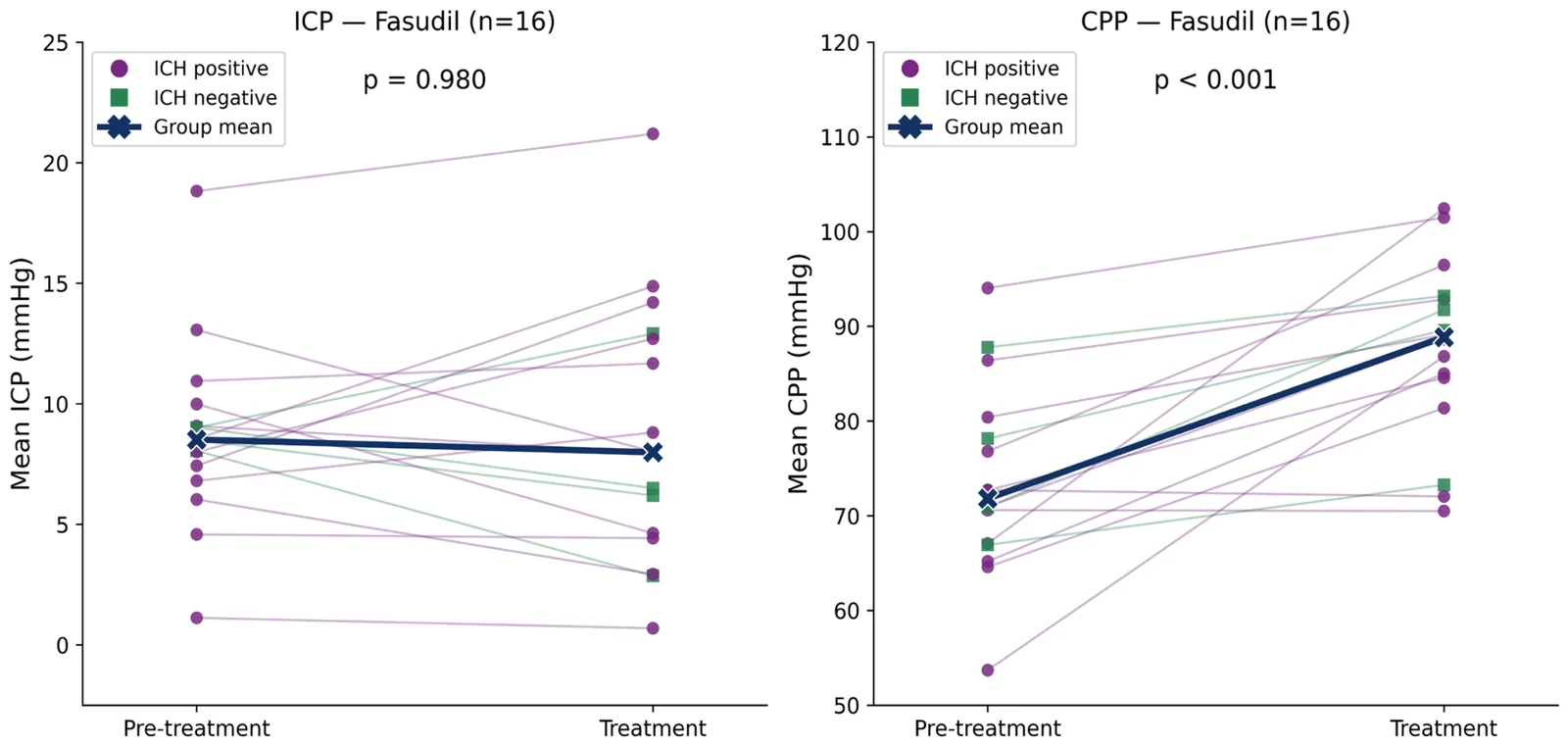
ICP and CPP changes between the pre-treatment and treatment periods (full fasudil treatment period; exploratory analysis). Individual changes in mean ICP (**left panel**) and CPP (**right panel**) between the pre-treatment and treatment periods (fasudil treatment period) in 16 patients. Given the small sample size, these findings are considered exploratory and should be interpreted with caution. Symbols and colors are as described in Figure 3. Statistical comparison was performed using the Wilcoxon signed-rank test.

In the fasudil group, 16 patients had sufficient pre-treatment and treatment-period data for analysis. The median ICP before fasudil initiation was 8.5 mmHg (IQR 7.3–9.3 mmHg) and during treatment was 8.0 mmHg (IQR 4.6–12.8 mmHg), with a median ΔICP of −0.3 mmHg (IQR −2.7 to +2.8 mmHg; 95% CI −2.31 to +2.27 mmHg; *p* = 0.980), indicating no significant change. A pattern of significant CPP elevation consistent with that observed in the clazosentan group was also present: median CPP increased from 71.8 mmHg (IQR 67.0–78.7 mmHg) to 88.9 mmHg (IQR 83.8–92.9 mmHg), with a median ΔCPP of +11.7 mmHg (IQR +6.4 to +19.7 mmHg; 95% CI +7.56 to +19.75 mmHg; *p* = 0.0002). Given the small sample size and exploratory nature of this analysis, these findings should be interpreted with caution (Figure 5).

### 3.6. ICP, CPP, and In-Hospital Mortality

**Figure 6:**
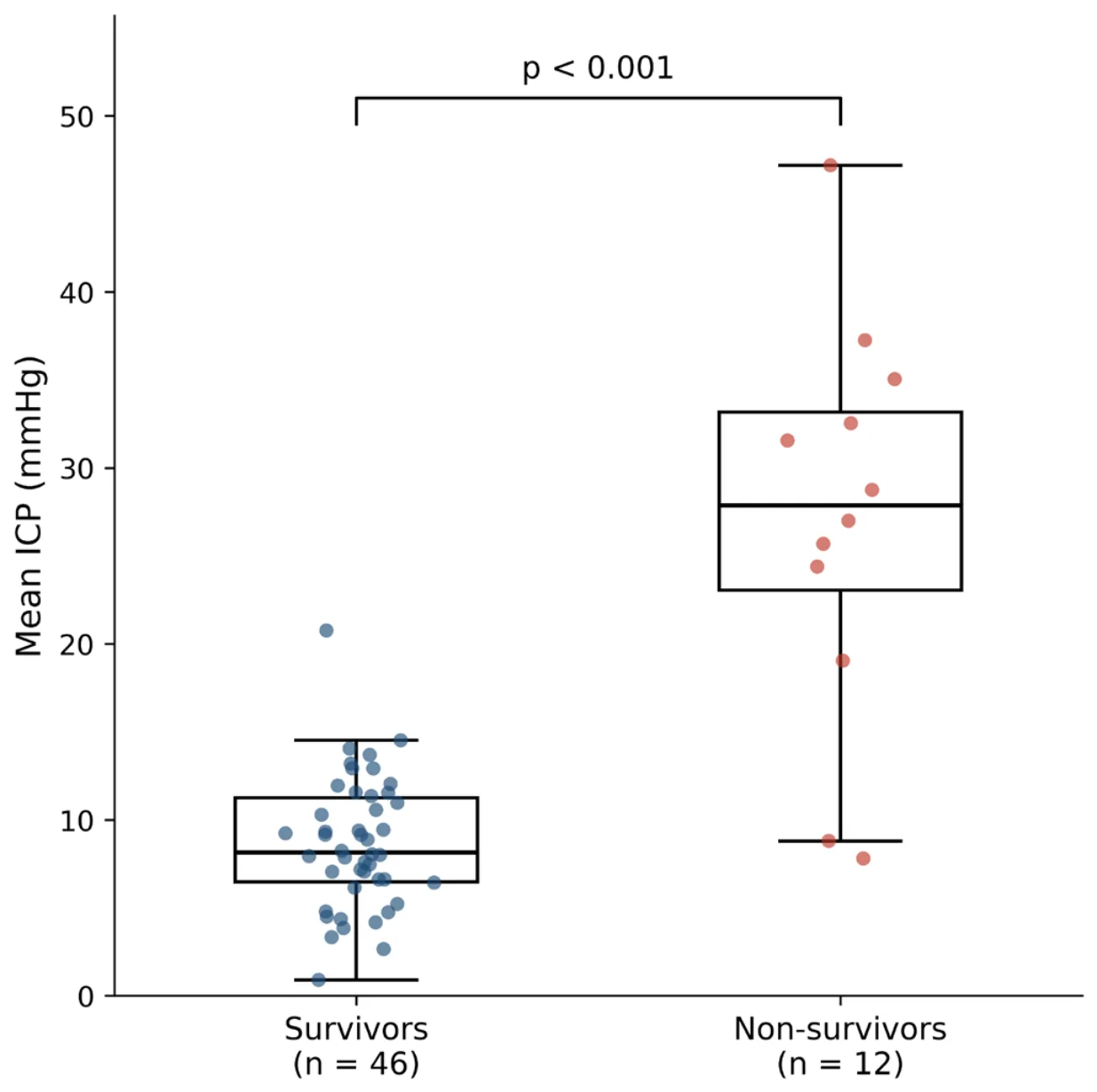
Mean ICP in survivors and non-survivors. Box plots with overlaid strip plots showing the distribution of mean ICP values in survivors (*n* = 46) and non-survivors (*n* = 12) among all ICP-monitored patients with available ICP data (*n* = 58). Non-survivors were defined as in-hospital death (modified Rankin Scale score = 6). The horizontal line within each box indicates the median; the box boundaries represent the interquartile range; whiskers extend to 1.5 times the interquartile range. Statistical comparison was performed using the Mann–Whitney U test. ICP, intracranial pressure; mRS, modified Rankin Scale.

Among all 59 ICP-monitored patients, 13 died during hospitalization (22.0%), of whom 11 were in the no vasospasm prophylaxis group. ICP data sufficient for analysis were available for 12 of these 13 patients. Among the 58 patients with analysable ICP data, non-survivors (*n* = 12) had a significantly higher median ICP than survivors (*n* = 46) (27.9 mmHg, IQR 23.1–33.2 vs. 8.1 mmHg, IQR 6.5–11.3; *p* < 0.001), and a significantly lower median CPP (42.7 mmHg, IQR 31.0–58.4 vs. 79.5 mmHg, IQR 74.0–85.3; *p* < 0.001) (Figure 6).

## 4. Discussion

Cerebral edema is a well-recognized complication of aneurysmal subarachnoid hemorrhage that occurs more frequently as disease severity increases [9]. The resultant elevation in intracranial pressure (ICP) is an established predictor of poor neurological outcome and mortality [10,11]. Because cerebral edema has also been reported during clazosentan treatment (6.0–13%) [3,7,8], concern has arisen that clazosentan-associated cerebral edema might further increase ICP, particularly in patients with poor-grade aSAH and concomitant intracerebral hemorrhage. This concern has likely discouraged the use of clazosentan in precisely those patients who may benefit most from it. In the present cohort, the majority of clazosentan-treated patients had poor neurological grades (95.7% with WFNS grade 4–5), representing the population in whom this concern is most clinically relevant.

Although cerebral edema is generally considered an important contributor to intracranial hypertension, ICP reflects the balance between intracranial volume and multiple compensatory mechanisms and therapeutic interventions, including cerebrospinal fluid displacement, venous blood redistribution, decompressive craniectomy, cerebrospinal fluid drainage, osmotherapy, sedation, mechanical ventilation, and temperature control. Therefore, cerebral edema alone does not necessarily translate into clinically significant ICP elevation, highlighting the importance of direct ICP monitoring during clazosentan administration.

Despite this concern, no statistically significant increase in ICP was observed during clazosentan administration. Across the full treatment period, the median ICP was 6.4 mmHg before treatment and 8.4 mmHg during treatment (median ΔICP +0.5 mmHg, 95% CI −1.74 to +2.64; *p* = 0.708). Similar findings were observed during the first 7 days after treatment initiation—corresponding to the period of greatest risk for fluid retention-related complications [4]—when the median ICP was 8.6 mmHg (median ΔICP +0.09 mmHg, 95% CI −1.78 to +2.59; *p* = 0.708). This finding was consistent in subgroups stratified by concomitant ICH: no significant ICP change was observed in either patients with ICH (median ΔICP −0.8 mmHg, 95% CI −4.89 to +1.55; *p* = 0.375) or those without ICH (median ΔICP +1.7 mmHg, 95% CI −1.72 to +4.55; *p* = 0.426). Notably, seven of the eight patients with ICH underwent craniotomy and six underwent decompressive craniectomy; their contribution to the absence of ICP elevation cannot be determined from this study. No patient in the clazosentan group developed a mean ICP exceeding 20 mmHg during monitoring (maximum individual mean, 14.8 mmHg).

These findings should be interpreted within the context of routine neurosurgical management. The present study was not designed to compare ICP between the clazosentan and fasudil groups, nor to isolate the pharmacological effect of clazosentan on ICP independent of concurrent ICP-directed therapies. Multiple interventions that directly influence ICP—including CSF drainage, decompressive craniectomy, hematoma evacuation, osmotherapy, sedation, mechanical ventilation, and blood pressure management—were applied according to institutional practice. These interventions were not systematically quantified and were not statistically adjusted for, and the absence of a statistically significant increase in ICP should not be interpreted as reflecting the isolated pharmacological effect of clazosentan. Rather, the present findings indicate that, in this retrospective cohort managed according to standard neurosurgical practice, no clinically significant ICP elevation was evident during clazosentan administration. To date, the only direct evidence on the effect of clazosentan on ICP has come from an experimental rat SAH model, in which clazosentan did not alter ICP under physiological conditions and did not exacerbate the acute ICP elevation following SAH induction [13]. To our knowledge, the present study is the first to extend these observations to the clinical setting by continuously monitoring ICP during clazosentan administration in aSAH patients; nevertheless, these findings must be interpreted with caution given the small, highly selected, and intensively managed nature of the cohort.

While ICP did not increase, CPP rose significantly during clazosentan administration, from a median of 69.1 mmHg before treatment to 84.5 mmHg during treatment (median ΔCPP +14.0 mmHg, 95% CI +8.93 to +17.44; *p* < 0.001). Since CPP is calculated as the difference between MAP and ICP, this elevation is consistent with an increase in MAP, although this could not be confirmed because MAP data were unavailable. A similar pattern was observed in the fasudil group, which is consistent with the possibility that the CPP elevation was not specific to clazosentan.

It is noteworthy that no in-hospital deaths occurred in the clazosentan group, in contrast to the no vasospasm prophylaxis group in which 11 of 12 patients (91.7%) died during hospitalization. This difference almost certainly reflects selection bias rather than a treatment effect: patients who were considered too critically ill to receive vasospasm-directed pharmacotherapy were concentrated in the no vasospasm prophylaxis group, representing a population with an extremely poor prognosis independent of any intervention. This observation underscores the inherent limitations of retrospective, non-randomized comparisons in this setting and should not be interpreted as evidence of a survival benefit of clazosentan.

The present study has several limitations. First, this was a single-center retrospective study involving a small, highly selected cohort of patients undergoing direct ICP monitoring. The sample size was limited, particularly for the exploratory fasudil analysis (*n* = 16). The study was therefore underpowered to detect small differences in ICP, and a post hoc power analysis demonstrated only 7.4% power to detect the observed effect size (r = 0.10) at *n* = 21. The possibility of a Type II error therefore cannot be excluded, and the absence of a statistically significant difference should not be interpreted as evidence of equivalence.

Second, the decision to place an ICP sensor was based on clinical judgment rather than predefined criteria, introducing potential selection bias, as monitored patients likely represented a more severely affected subset of patients with aSAH. Because nearly all clazosentan-treated patients had poor-grade aSAH (WFNS grade 4–5) and underwent ICP monitoring according to institutional practice, these findings cannot be generalized to good-grade aSAH patients, to patients who do not undergo ICP monitoring, or to centers with different neurocritical care protocols.

Third, although the fasudil group provided an exploratory comparator, the study was not designed for formal between-group comparison. Treatment allocation was inherently non-random: clazosentan was preferentially administered after its approval in April 2022, whereas fasudil was used in patients considered less suitable for clazosentan and over a substantially longer study period (2010–2025 vs. 2023–2025). Consequently, the two cohorts should not be considered directly comparable.

Fourth, interventions that may influence ICP were not incorporated into the statistical analysis. Although adjustment using a linear mixed-effects model would have been methodologically preferable, these interventions were not systematically quantified in this retrospective dataset, and the limited sample size would not have supported such an analysis. Therefore, the observed findings should not be interpreted as reflecting the isolated pharmacological effect of clazosentan.

Fifth, the before–after study design is inherently susceptible to temporal confounding. Consequently, the absence of a significant change in ICP may partly reflect the natural course of aSAH rather than the effect of clazosentan itself.

Sixth, serial MAP data were not formally analyzed. Consequently, the mechanism underlying the observed increase in CPP could not be directly verified, and the proposed explanation remains speculative.

## 5. Conclusions

In this retrospective single-center cohort, no statistically significant ICP elevation was observed during clazosentan administration, either across the full treatment period or during the first 7 days after initiation. This finding was consistent regardless of concomitant ICH, and no patient had a mean ICP exceeding 20 mmHg during treatment. However, these findings should be interpreted cautiously, as the small sample size limits statistical power and the possibility of a false-negative result cannot be excluded. Moreover, given that this cohort consisted almost exclusively of patients with poor-grade aSAH undergoing direct ICP monitoring and managed according to a single-center neurocritical care protocol, these findings should not be generalized to patients with good-grade aSAH, those who do not undergo ICP monitoring, or centers with different neurocritical care protocols. Nevertheless, to our knowledge, this is the first report to directly examine the effect of clazosentan on ICP using continuous direct ICP monitoring, and these findings may help inform clinical decision-making regarding the use of clazosentan in patients with poor-grade aSAH, pending confirmation in larger prospective studies.

## Figures and Tables

**Table 1:** Baseline characteristics of patients with aneurysmal subarachnoid hemorrhage who underwent direct ICP monitoring.

Variable	Clazosentan (*n* = 23)	Fasudil (*n* = 24)	No Vasospasm Prophylaxis (*n* = 12)	*p*-Value * (C vs. F)
**Demographics**				
Age, years, median (IQR)	66 (58–74)	70 (54–76)	66 (54–74)	0.725
Female sex, *n* (%)	16 (69.6)	17 (70.8)	10 (83.3)	1.000
**Clinical severity**				
WFNS grade, *n* (%)				
1	1 (4.3)	2 (8.3)	0 (0)	—
2	0 (0)	1 (4.2)	1 (8.3)	—
3	0 (0)	3 (12.5)	0 (0)	—
4	11 (47.8)	5 (20.8)	1 (8.3)	—
5	11 (47.8)	13 (54.2)	10 (83.3)	—
WFNS grade 4–5, *n* (%)	22 (95.7)	18 (75.0)	11 (91.7)	0.097
ICH, *n* (%)	8 (34.8)	16 (66.7)	4 (33.3)	0.042 †
**Surgical treatment**				
Surgical clipping, *n* (%)	12 (52.2)	21 (87.5)	4 (33.3)	0.011 †
Endovascular treatment, *n* (%)	11 (47.8)	3 (12.5)	8 (66.7)	—
Decompressive craniectomy, *n* (%)	7 (30.4)	10 (41.7)	3 (25.0)	0.547
CSF drainage (EVD or SD), *n* (%)	20 (87.0)	16 (66.7)	9 (75.0)	0.168
**Drug administration**				
Drug start, days, median (IQR)	2 (2–3)	4 (3–4)	—	0.001 †
Treatment duration, days, median (IQR)	12 (12–13)	11 (10–12)	—	0.052
**Outcome**				
mRS 0–2, *n* (%)	7 (30.4)	4 (16.7)	0 (0)	0.318
In-hospital mortality, *n* (%)	0 (0)	2 (8.3)	11 (91.7)	0.489

Data are presented as median (interquartile range) or *n* (%). * *p*-values for comparisons between the clazosentan and fasudil groups only; the no vasospasm prophylaxis group was not included in formal comparisons. † Statistically significant (*p* < 0.05). Abbreviations: IQR, interquartile range; WFNS, World Federation of Neurological Surgeons; ICH, intracerebral hemorrhage; CSF, cerebrospinal fluid; EVD, external ventricular drainage; SD, spinal drainage; DAY, days after aSAH onset (onset = day 0); mRS, modified Rankin Scale.

## Data Availability

The raw data supporting the conclusions of this article will be made available by the author upon reasonable request.
